# Foramen Tympanicum (Foramen of Huschke) as a Cause of Unexplained Spontaneous Otorrhea: A Case Report

**DOI:** 10.7759/cureus.66658

**Published:** 2024-08-11

**Authors:** Dimitar Pazardzhikliev, Maria Kraeva, Petar Uchikov

**Affiliations:** 1 Department of Otorhinolaryngology, Faculty of Medicine, Medical University of Plovdiv, Plovdiv, BGR; 2 Department of Otorhinolaryngology, Kaspela University Hospital, Plovdiv, BGR; 3 Department of Special Surgery, Faculty of Medicine, Medical University of Plovdiv, Plovdiv, BGR

**Keywords:** amylase, otalgia, otorrhea, spontaneous salivary fistula, foramen of huschke, foramen tympanicum

## Abstract

The foramen of Huschke or foramen tympanicum is a developmental defect in the bony part of the external auditory meatus. It occurs due to incomplete obliteration of the tympanic canal and the persistence of openings along the canal. This leads to the presence of a salivary fistula and the most common symptoms of the condition are otalgia, otorrhea, and hearing loss. We report a case of a 69-year-old female patient who presented to the ENT with symptoms of watery discharge from the left ear for 12 years. In the last two weeks, pain and itching appeared. Otoscopy showed the presence of a clear fluid in the external auditory meatus with a normal tympanic membrane. Biochemical analysis of the fluid proved the presence of amylase. Computed tomography showed a fistula between the parotid gland and the external ear canal. The diagnosis of the persistent foramen of Huschke remains difficult due to the fact that it could mimic many other conditions. The otalgia and otorrhea are symptoms that suggest a very wide differential diagnosis. Misdiagnosis and inappropriate treatment are common.

## Introduction

The foramen of Huschke or foramen tympanicum is a developmental defect in the antero-inferior part of the bony external auditory meatus [1]. It was first described by Sharma et al. back in 1984 [2]. The defect is due to incomplete obliteration of the tympanic canal and the persistence of openings along the canal that occur in embryonic development during bone formation. This leads to the presence of a spontaneous salivary fistula. The pathology is rare. According to some authors, the prevalence is between 2% and 23% [3]. In the majority of the cases, it is unilateral. The most common symptoms are otalgia, otorrhea, hearing loss, and tinnitus [4]. In this article, we aim to discuss a case of persistent foramen tympanicum.

## Case presentation

A 69-year-old female with no co-morbidities presented to the ENT department with the complaint of clear watery discharge from the left ear for 12 years. The spontaneous secretion filled the canal during feeding. No pain was present. No history of trauma was present. In the last two years, the patient noticed an increase in the discharge. In the last week, severe pain and itching appeared.

Physical examination of the left ear revealed the presence of a narrowed external auditory canal and the presence of a homogeneous clear fluid in the same. On the front wall of the bony part of the canal, a fistulous opening was visible, from which a clear secretion flowed. During the act of chewing, the secreted secretion increased and almost completely filled the canal. The tympanic membrane was intact (Video 1). No pathology of the right ear was detected during otoscopy. No pathology of the nose and throat was found.

**Video 1:** Endoscopy using a zero-degree endoscope showing the presence of a homogeneous clear fluid. A fistulous opening is visible.

Blood tests were as follows: WBC of 8.18 g/L, RBC of 4.34/L, HGB of 135.0 g/L, PLT of 250.0/L, and fibrinogen of 2.9 g/L. Computed tomography of the temporo-mandibular joints (TMJ), parotid glands, larynx, and neck was performed. It revealed the normal morphology of the structures. A bony defect between the left TMJ and the external acoustic canal (EAC) was present.

The ear discharge was collected in a container and sent for biochemical analysis. Results showed the presence of salivary amylase. Persistent foramen of Huschke was confirmed.

**Figure 1:**
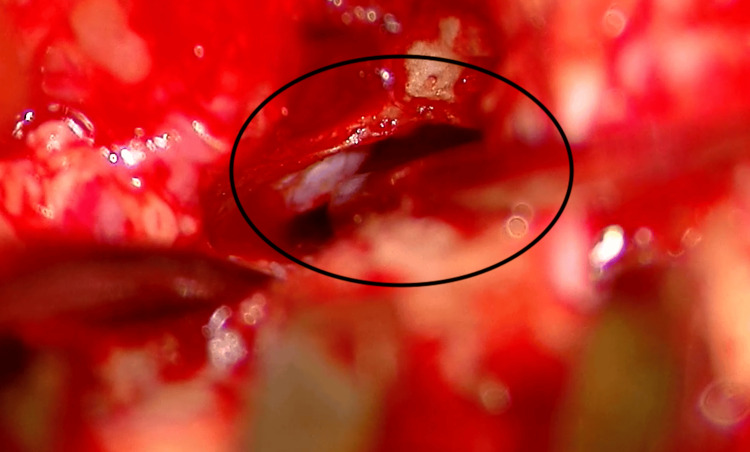
Intraoperative finding of a soft tissue tract.

The patient underwent surgical treatment under general anesthesia. A preauricular incision was made, the subcutaneous tissue was removed, and the EAC and parotid gland were reached. A soft tissue tract was present in the area between the canal and the gland (Figure 1).

The presence of the fistula was confirmed by using methylene blue ink. The color of the soft tissue tract changed after the ink was inserted in the EAC. The fistula was ligated, and the defect was filled with a cartilage graft, which was harvested from the tragus. External auditory canal tamponade was performed. The patient's postoperative period was uneventful, and she was discharged on the third day. An oral broad-spectrum antibiotic was prescribed. The tamponade was removed after two weeks. After removal, there was no evidence of leakage.

The patient was followed up for one year after the surgery, and there was no evidence of ear discharge from the ear.

## Discussion

Foramen tympanicum or foramen of Huschke is a defect in the development of the tympanic bone. van der Meer et al. concluded that the foramen tympanicum has been described in relation to TMJ herniations, external ear canal salivary fistulas, tumor spread, or drainage of odontogenic abscesses in the ear canal [5]. According to Tozoglu et al., the foramen of Huschke could predispose individuals to the pathology of the temporomandibular joint, or it could be associated with saliva discharge in the ear canal during mastication [1]. As stated by Rana et al., the embryonic development of the external auditory canal begins around the eighth week of gestation. The tympanic ring plays an important role in the development of the tympanic bone. It forms four centers of ossification, which merge and form the bone. This process begins at nine weeks of gestation. Incomplete fusion results in the presence of a dehiscence, which is located between the bony portion of the canal and medial to the temporomandibular joint [6]. This results in the presence of spontaneous salivary fistula. Its prevalence is between 2% and 23% according to Kinar et al. [3]. A radiologically patent foramen is not necessarily symptomatic.

Sharma et al. first reported a case of a 58-year-old patient with a patent foramen of Huschke [2]. Rana et al. reported a case of a bilateral salivary fistula in an eight-year-old child. They performed a two-stage treatment, first operating on one ear, and four months later on the other. They used a temporalis fascia graft [6]. In a case described by Tasar et al., a congenital salivary fistula was present, along with chronic sialadenitis and parotid cyst in a 21-year-old male patient. They performed an excision of the cyst with partial parotidectomy and closure of the persistent foramen of Huschke with tragal cartilage and temporalis fascia [7].

According to Macielak et al., the main presenting symptom is otalgia, followed by otorrhea and subjective hearing loss [4]. The main presenting symptom in our case was otorrhea, which was present for 12 years prior to hospitalization. Pain and itching appeared in the last two weeks.

The discharge in cases of a patent foramen of Huschke does not have a color or a smell. A bad smell could indicate a more severe condition. Proper investigation and subsequent management are very important because the differential diagnosis in cases of ear discharge is very wide. As stated by Rana et al., misdiagnosis and inadequate treatment are common. This is due to the fact that this condition is rare and nonspecific [6].

Decisive for the diagnosis is the biochemical analysis of the discharge fluid. It proves the presence of amylase in it. This is decisive for the pre-operative assessment because it helps us distinguish between a salivary fistula and synovial fluid leak [8,9].

Imaging studies are also essential for the diagnosis. The foramen tympanicum is well visible on a CT scan because it provides the best detail for bony anatomy. MRI, scintigraphy, and sialography are also helpful. Tozoglu et al. used cone-beam computer tomography (CBCT) to demonstrate the foramen tympanicum [1].

As stated by Teoh et al., the management of the condition depends on the size of the defect, the severity of the symptoms, and the patient's preferences. Surgical treatment is of utmost importance for closing the dehiscence. Besides open approaches, a transcanal endoscopic approach has also been reported [10]. Open approaches could be performed with preauricular or endaural incision and different types of graft materials are being used in order for the dehiscence to be closed [11,12]. The endoscopic approach uses an endoscope to raise a skin flap and a graft for the dehiscence repair. It is considered less invasive [13].

## Conclusions

Diagnosing a persistent foramen of Huschke (foramen tympanicum) remains a challenge because it can often mimic other conditions. Ear pain and ear discharge are symptoms that have a wide differential diagnosis. Actively seeking and considering the presence of a fistula is necessary. Biochemical analysis of the fluid, as well as imaging studies such as CT, CBCT, MRI, and sialography, are key to making the diagnosis.

## References

[1] Tozoglu U, Caglayan F, Harorli A (2012). Foramen tympanicum or foramen of Huschke: anatomical cone beam CT study. Dentomaxillofac Radiol.

[2] Sharma PD, Dawkins RS (1984). Patent foramen of Huschke and spontaneous salivary fistula. J Laryngol Otol.

[3] Kinar A, Bucak A, Ulu Ş (2020). An otalgia cause: temporomandibular joint herniation from foramen of Huschke to external auditory canal. J Craniofac Surg.

[4] Macielak RJ, Nassiri AM, Fillmore WJ, Lane JI, Driscoll CL, Carlson ML (2022). Persistent foramen of Huschke: presentation, evaluation, and management. Laryngoscope Investig Otolaryngol.

[5] van der Meer WL, van Tilburg M, Mitea C, Postma AA (2019). A persistent foramen of Huschke: a small road to misery in necrotizing external otitis. AJNR Am J Neuroradiol.

[6] Rana K, Rathore PK, Raj A, Meher R, Wadhwa V, Prakash A, Rajan S (2015). Bilateral spontaneous salivary otorrhoea: case report and a review of the literature. Int J Pediatr Otorhinolaryngol.

[7] Tasar M, Yetiser S (2003). Congenital salivary fistula in the external auditory canal associated with chronic sialoadenitis and parotid cyst. J Oral Maxillofac Surg.

[8] Langer J, Begall K (2004). [Otosialorrhoea - diagnostics and therapy of a salivary fistula of the external auditory canal]. Laryngorhinootologie.

[9] Chilla R (2002). [Otosialorrhoea - a rare case of a spontaneous salivary fistula of the external auditory canal]. HNO.

[10] Teoh T, Abdullah A, Kumarasamy G (2024). Finding the silver bullet for persistent foramen Hushke. Cureus.

[11] Yoo MH, Park JW, Lee HS, Yang CJ, Park HJ (2016). Repair of the foramen of Huschke using an extended endaural approach. Laryngoscope.

[12] Al-Kayat A, Bramley P (1979). A modified pre-auricular approach to the temporomandibular joint and malar arch. Br J Oral Surg.

[13] Xie B, Zhang S, Liu Y (2019). Endoscopic-assisted repair of spontaneous temporomandibular joint herniation through a transcanal approach. Otol Neurotol.

